# Ground penetrating radar observations of the contact between the western delta and the crater floor of Jezero crater, Mars

**DOI:** 10.1126/sciadv.adi8339

**Published:** 2024-01-26

**Authors:** David A. Paige, Svein-Erik Hamran, Hans E. F. Amundsen, Tor Berger, Patrick Russell, Reva Kakaria, Michael T. Mellon, Sigurd Eide, Lynn M. Carter, Titus M. Casademont, Daniel C. Nunes, Emileigh S. Shoemaker Thackston, Dirk Plettemeier, Henning Dypvik, Sanna Holm-Alwmark, Briony H. N. Horgan

**Affiliations:** ^1^ University of California, Los Angeles, Los Angeles, CA, USA.; ^2^ University of Oslo, Oslo, Norway.; ^3^ Vestfonna Geophysical, Trondheim, Norway.; ^4^ Cornell University, Ithaca, NY, USA.; ^5^ University of Arizona, Tucson, AZ, USA.; ^6^ Jet Propulsion Laboratory, California Institute of Technology, Pasadena, CA, USA.; ^7^ Technische Universität Dresden, Dresden, Germany.; ^8^ Lund University, Lund, Sweden.; ^9^ Purdue University, West Lafayette, IN, USA.

## Abstract

The delta deposits in Jezero crater contain sedimentary records of potentially habitable conditions on Mars. NASA’s Perseverance rover is exploring the Jezero western delta with a suite of instruments that include the RIMFAX ground penetrating radar, which provides continuous subsurface images that probe up to 20 meters below the rover. As Perseverance traversed across the contact between the Jezero crater floor and the delta, RIMFAX detected a distinct discontinuity in the subsurface layer structure. Below the contact boundary are older crater floor units exhibiting discontinuous inclined layering. Above the contact boundary are younger basal delta units exhibiting regular horizontal layering. At one location, there is a clear unconformity between the crater floor and delta layers, which implies that the crater floor experienced a period of erosion before the deposition of the overlying delta strata. The regularity and horizontality of the basal delta sediments observed in the radar cross sections indicate that they were deposited in a low-energy lake environment.

## Teaser

Subsurface radar imaging reveals a history of erosion of the Jezero crater floor followed by the deposition of lake sediments.

## INTRODUCTION

NASA’s Mars Perseverance rover is exploring the western edge of Jezero crater, a ~50-km impact crater that has been interpreted to be a Noachian-aged lake basin bordered by remnant fluvial delta deposits (*1*–*8*). Before landing, the stratigraphic relationships between the presently exposed crater floor units and the Jezero western delta had not been definitively determined. On the basis of orbital mapping and spectroscopic data, most previous studies concluded that Jezero western delta deposits were embayed by younger, dark-toned crater floor units of volcanic origin (*3*, *4*, *9*). However, a more recent analysis favors the conclusion that Jezero western delta lies stratigraphically above all the crater floor deposits (*7*). Determining whether the delta deposits are older or younger than the crater floor is fundamental to understanding the overall geologic and hydrologic history of Jezero crater, as well as the Perseverance rover samples.

**Fig. 1.**
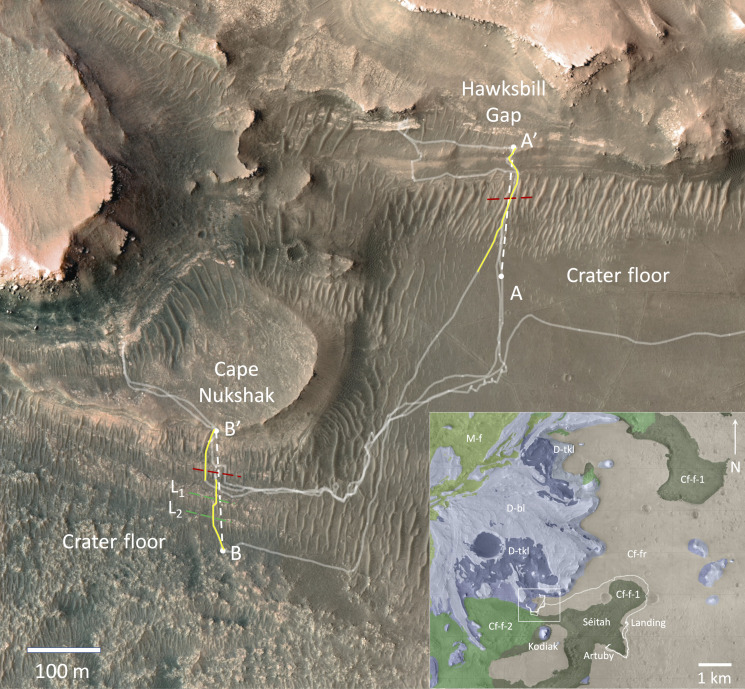
Orbital context maps of the Jezero western delta front region. Orbital High Resolution Imaging Science Experiment (HiRISE) (*28*) color base map showing the path (pale white lines) of the Perseverance rover across the crater floor and delta front through Sol 706, including contact-crossings at Hawksbill Gap and Cape Nukshak. The white dashed lines show the locations of the projected radar cross sections shown in Figs. 3 and 4. The solid yellow lines show the original pre-projected tracks, the red dashed lines indicate the inferred locations of the crater-floor delta contact from RIMFAX data, and the green dashed lines are the traces of two outcropping crater floor layers (L_1_ and L_2_) that can be traced to subsurface radar reflectors (See Fig. 4). The regional insert map shows the rover path since landing as well as the exposed regional bedrock geologic units mapped from pre-landing orbital imagery and spectroscopy (*13*). Cf-f-1, crater floor fractured 1; Cf-f-2, crater floor fractured 2; Cf-fr, crater floor fractured rough; D-bl, delta blocky; D-tkl, delta thickly layered; M-f, margin fractured.

Between 10 May and 8 December 2022, the Perseverance rover traversed across the contact between the crater floor and the Jezero western delta near Hawksbill Gap and near Cape Nukshak (Fig. 1), acquiring a set of RIMFAX ground penetrating radar soundings at 10-cm intervals along the traverse (*10*).

## RESULTS

**Fig. 2.**
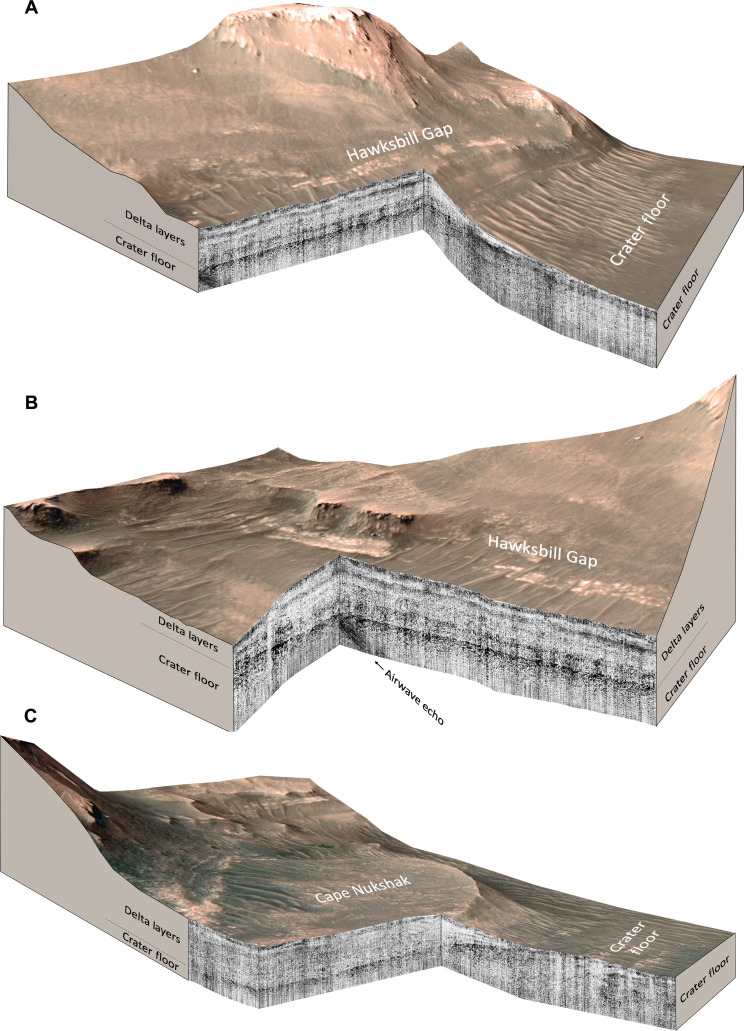
Cross-sectional projected 3D views of the RIMFAX reflection amplitude radargrams. (**A**) Hawksbill Gap acquired on Sols 535 to 541 and (**B**) Hawksbill Gap on Sols 449 to 460 and Sols 535 to 537. (**C**) Cape Nukshak on Sols 420 to 421 and Sols 556 to 632. Strongly reflecting layers are rendered as dark whereas weakly reflecting layers are rendered as light (see Materials and Methods). All views use HiRISE digital elevation model data and are 2× vertically exaggerated.

**Fig. 3.**
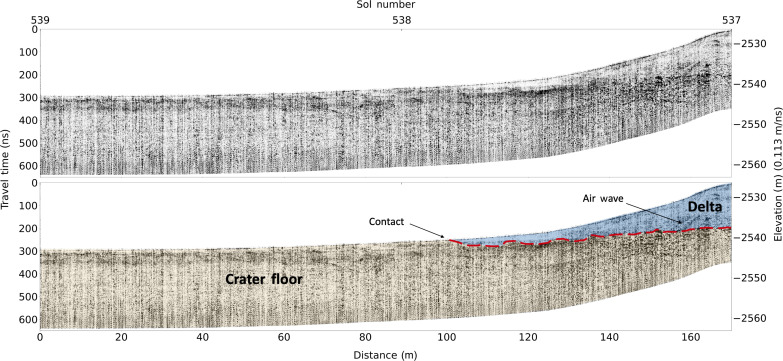
RIMFAX projected radargrams of the crater floor–delta contact at Hawksbill Gap. Unannotated (top) and annotated (bottom) RIMFAX Sol 538–539 projected reflection amplitude radargrams across the crater floor–delta contact at Hawksbill Gap (A-A′ in Fig. 1) with no vertical exaggeration.

**Fig. 4.**
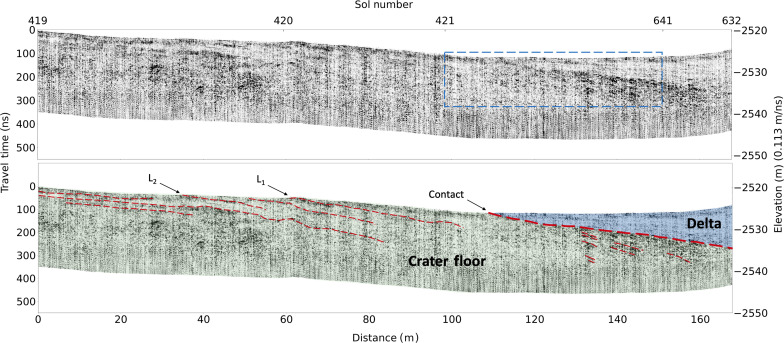
RIMFAX projected radargrams of the crater floor–delta contact at Cape Nukshak. Unannotated (top) and annotated (bottom) RIMFAX Sol 420–422 and Sol 565–566 projected reflection amplitude radargrams across the crater floor–delta contact at Cape Nukshak (B-B′ in Fig. 1) with no vertical exaggeration. The blue box shows the approximate boundaries of the enlarged region in Fig. 5, and L_1_ and L_2_ are two subsurface layers that outcrop in HiRISE orbital images (see Fig. 1).

**Fig. 5.**
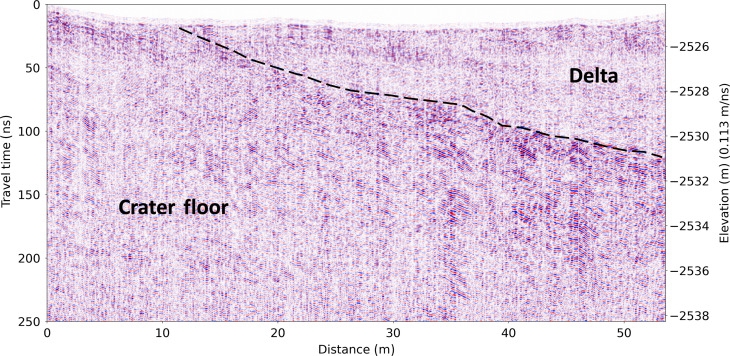
High-resolution projected RIMFAX radargram at Cape Nukshak. High-resolution Sol 641 radargram of the crater floor–delta contact at Cape Nukshak with 2× vertical exaggeration.

RIMFAX radargrams (Figs. 2 to 5) reveal aspects of the structure of the subsurface to depths of ~20 m below the surface (see Materials and Methods).

### Radar interpretation

RIMFAX observes a strong and consistent radar reflector that extends beneath the Jezero western delta in both the Hawksbill Gap and the Cape Nukshak sections that we interpret to be the crater floor–delta contact (Figs. 2 to 4). At Hawksbill Gap, the contact is approximately horizontal, whereas, at Cape Nukshak, the contact dips toward the north at an apparent dip angle of ~11° measured in the north-south direction, with an estimated true downward dip of ~12° measured in the north-northeast direction at 20° azimuth, which was determined in conjunction with surface delta–crater floor contact orientation. In both locations, the contact is overlain by a series of relatively horizontal reflectors that truncate at the exposed delta face at Hawksbill Gap, and at the contact itself at Cape Nukshak, which are interpreted to be strata of the lower delta. The basal delta layers observed in these radar profiles are generally less strongly reflecting than the top surface of the crater floor units, and show consistent horizontal layering structures over the full extent of the observations reported here, which cover ~100 m in the north-south and east-west directions.

In the Hawksbill Gap section (Figs. 2, A and B, and 3), there is discontinuous non-horizontal layering evident within the crater floor unit as well as zones of low radar reflectivity in the near-surface that appear to be analogous to crater floor radar facies observed by RIMFAX along the Artuby ridge (Fig. 1) earlier in the mission (*11*). In the vicinity of Hawksbill Gap, the crater floor units exposed on the surface are mapped as belonging to the Máaz formation based on orbital and rover data (*12*), which corresponds to the Cf-fr unit mapped in Fig. 1. However, we were not able to interpret the subsurface structures observed in the crater floor unit here to be uniquely associated with either the Máaz formation or the undifferentiated smooth surficial unit (*7*, *13*). At Hawksbill Gap, the surface expression of the crater floor–delta contact is obscured by regolith and eolian deposits. At the foot of the delta, RIMFAX observes a <3-m-thick weakly reflecting horizontally layered surface unit that is underlain by a bumpy strongly reflecting layer that continues northward under the delta (Fig. 3). We interpret the weakly reflecting near-surface unit to be a thin layer of uneroded basal delta sediments because of its association with subsurface delta sediments to the north, and the fact that RIMFAX did not observe similar layered surface deposits elsewhere on the crater floor. The bumpy strongly reflecting subsurface unit can be interpreted to represent the crater floor because of its continuity with crater floor units to the north and the south. This interpretation places the surface boundary between the delta and the crater floor approximately 30 m south of where previously mapped (*13*). This boundary location is compatible with the gradational compositional boundary mapped between the high-calcium pyroxene-dominated units of the Máaz formation and the low-calcium pyroxene-dominated units of the lower delta in this area based on MRO CRISM data (*14*).

In the Cape Nukshak section south of the delta (Figs. 2C and 4), there are a series of strongly reflecting layers within the crater floor unit that dip downward to the north at angles of ~4° to 8°. South of the delta, these layers are truncated at the surface and appear to correspond to a set of exposed northwest-southeast trending light-toned layers that are visible from orbit that we designate L_1_ and L_2_ (Fig. 1). In the vicinity of Cape Nukshak, the crater floor units exposed on the surface are mapped as belonging to the Cf-f-2 unit (Fig. 1), which is compositionally related to Séitah (*12*). Beneath the delta, RIMFAX high-resolution radargrams reveal a larger number of closer spaced similarly dipping layers within the crater floor unit that appear truncated at the sloping crater floor–delta contact (Fig. 5). The location of the intersection between crater floor and delta units determined by RIMFAX is consistent with the compositional boundary mapped at lower resolution from orbit between the olivine-dominated Cf-f-2 unit and the low-calcium pyroxene-dominated units of the delta (*14*).

On the basis of RIMFAX propagation velocity measurements, the average density of the lower delta sediments is estimated to be 2.84 g cm^−3^ (see Materials and Methods) compared to a value of 3.1 g cm^−3^ which was derived from earlier RIMFAX measurements for the crater floor (*15*).

### Geologic interpretation

The superposition relationships observed by RIMFAX in the vicinity of the delta front at both locations demonstrate that the Jezero western delta sediments are younger than the crater floor. In the Cape Nukshak section, the top surface of the crater floor unit is sloped and uneven, and underlies a succession of onlapping basal delta layers that truncate at the contact boundary, creating a clear unconformity. On the basis of accepted geological definitions, if the underlying crater floor here is sedimentary, then the structure would be classified as an angular unconformity, whereas if the underlying crater floor here is metamorphic or igneous, then the structure would be classified as a nonconformity (*16*). While it is generally acknowledged that the majority of the material that has filled Jezero crater is sedimentary (*3*), analyses of in situ crater floor observations by Perseverance that have been published to date have interpreted both the Máaz and Séitah formations to be most likely of igneous origin (*8*, *12*, *17*). Near Artuby ridge, RIMFAX observed a series of strongly reflecting layers that dipped away from the boundary Séitah (*11*). The L_1_ and L_2_ crater floor layers (Figs. 1 and 4) also dip away from the core of the Cf-f-2 unit and the Séitah formation, which is consistent with the inference that the crater floor units in the vicinity of Séitah have a generally domical structure (*8*), However, the origin of this structure and the processes responsible are not known (*8*).

The presence of an unconformity at the crater floor–delta contact at Cape Nukshak requires that the crater floor experienced a period of erosion before the deposition of the basal delta sediments. The cause(s) and extent of this erosional episode cannot be determined uniquely from the observations reported here nor can the amount of time that elapsed between these events be constrained. Earlier in the mission in the Artuby region, RIMFAX observed a set of strongly reflecting dipping subsurface layers in the crater floor that were truncated at the surface, implying that at least several meters of erosion of the crater floor had taken place (*11*). The erosional episode that exposed the Artuby ridge outcrops may have occurred synchronously with the erosional episode implied by the discordant crater floor–delta contact at Cape Nukshak or it may have occurred during a later period of erosion subsequent to the deposition of the basal delta succession. The relative horizontality of the crater floor–delta contact at Hawksbill Gap compared to Cape Nukshak may owe in part to the presence of the Máaz formation, which overlies and postdates the Séitah formation and tends to be unusually smooth in the area adjacent to Hawksbill Gap (*12*). The strong radar reflections observed at the top of the crater floor units at both locations indicate a change in dielectric constant that could be associated with the presence of a physical or chemical weathering horizon that could have developed before the deposition of overlying sediments.

The horizontal layering observed by RIMFAX in the basal delta units at Hawksbill Gap and Cape Nukshak is most easily explained by the cyclic deposition of sediments in a relatively low-energy aqueous environment. At Hawksbill Gap, the horizontal layers terminate at the exposed delta face with no evidence of slumping. At Cape Nukshak, the onlapping geometry of the basal delta succession relative to the sloping crater floor surface is characteristic of depositional environments in which sediments are deposited on pre-existing rising distal basal topography (*18*). Because the spatial extent of the observations reported here only covers a small fraction of the Jezero crater floor and delta system, it is difficult to determine whether these basal delta layers represent a transgressive sequence of flat-lying lakebed sediments that existed before the formation of the delta or whether they represent bottomset beds associated with an aggrading delta deposit to the northwest. The basal layers may have also experienced compaction and deformation driven by overburden pressure and the loss of pore-filling fluids (*19*).

## DISCUSSION

The stratigraphic relationships we have identified based on the RIMFAX observations of the crater floor–delta contact allow us to form a clearer picture of the portion of martian history that they record. A number of previous authors have suggested that the Jezero western delta once extended considerably further onto the crater floor before eroding back to its present boundaries (*2**–**5*, *13*, *20*). The observation of the presence of a succession of horizontal layers at the base of the delta at two separate locations, and their exposure due to erosion at the delta front, supports this notion. While we cannot directly estimate how far onto the crater floor these layers once extended, they point to the past existence of a low-energy sedimentary deposition zone that could have extended out at least as far as the isolated delta-associated deposits found more than 7 km beyond the present location of the delta front (*20*).

The existence of horizontally bedded sediments on the floor of Jezero crater can place constraints on the hydrologic environments in which they were deposited. In subaerial lacustrine environments, widespread low-relief sedimentary structures such as those observed here are associated with deposition in underfilled (evaporative) or balance-filled (fluctuating) lake systems, whereas high-relief sedimentary structures with sharply dipping layers are associated with overfilled (overflowing) lake systems (*3*, *21*). Low-energy sedimentary environments may also be found at the bottoms of subglacial lakes (*22*), which may have existed beneath a thick ice sheet that has been hypothesized to cover all of Isidis Planitia and Nili Fossae 3 billion years ago (*23*).

Early Perseverance imaging observations of the upper delta revealed the presence of extensive boulder deposits exposed on eroded scarps, suggesting a period of higher-energy fluvial deposition postdating the period of lower-energy fluvial deposition recorded in the basal delta sediments (*24*). The presence of these erosionally resistant rockier layers in the upper delta may have protected the less resistant finer-grained lower delta sediments beneath them from erosion through time (*2*), which provides a natural explanation for why thick sections of lower delta sediments are only observed today in association with the delta scarp and the Kodiak butte, which is interpreted to be an erosional remnant of the delta (*8*, *24*).

**Fig. 6.**
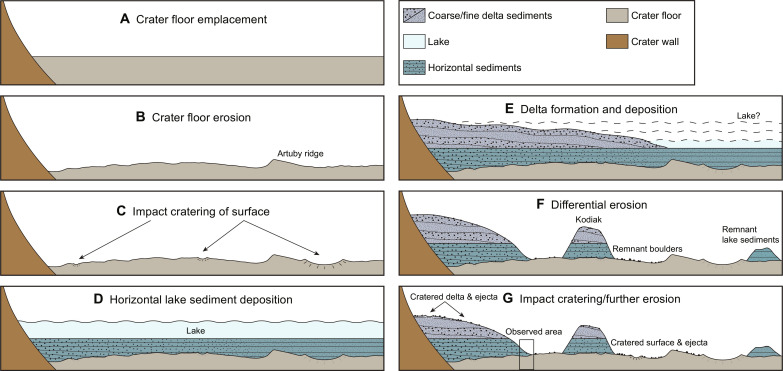
Diagrammatic reconstruction of the depositional and erosional history of the Jezero western delta region. (**A**) Crater floor emplacement. (**B**) Crater floor erosion. (**C**) Impact cratering of surface. (**D**) Horizontal lake sediment deposition. (**E**) Delta formation and deposition. (**F**) Differential erosion. (**G**) Impact cratering/further erosion.

RIMFAX measurements of the crater floor–delta contact have confirmed a number of aspects of the geological history of the western delta region of Jezero crater that have been proposed in previous studies (*1**–**7*, *13*, *20*, *24*) while adding some key pieces of information which we summarize in Fig. 6. A set of generally horizontal sediments were laid down onto a previously eroded and cratered crater floor unit. This first succession of horizontal sediments was laid down in a low-energy aqueous environment that likely extended well beyond the present location of the delta scarp. Subsequent layers of upper delta sediments were then deposited onto these basal sediments during a period of varying lake levels (*24*), and both were differentially eroded and cratered, leaving the horizontal basal sediments exposed beneath a protective upper delta succession. This sequence of events, which now appears to involve two distinct periods of sediment deposition sandwiched between two periods of erosion, reinforces the notion that Jezero crater has recorded a rich geological history that was driven by large-scale changes in the martian environment. A more complete understanding of these events may need to await the return and analysis of the Perseverance samples (*25*).

## MATERIALS AND METHODS

### Planetary data system dataset and processing

All original data used in this study are available through the NASA Planetary Data System Geoscience Node https://pds-geosciences.wustl.edu/missions/mars2020/rimfax.htm (*25*) and were processed as described in previous publications (*10*, *11*, *26*). Figures 2 to 4 show amplitude-normalized radargrams in black and white, where black indicates high reflectivity values and white indicates low reflectivity values. The reflectivity value is the magnitude of the received radar signal. In Fig. 5, the red and blue colors show the real value of the received radar signal, with red indicating a positive radar signal and blue indicating a negative radar signal. The negative elevation values on the *y* axes in Figs. 3 to 5 are relative to the MGS MOLA reference equipotential surface (*27*).

### Permittivity/wave velocity estimation

**Fig. 7.**
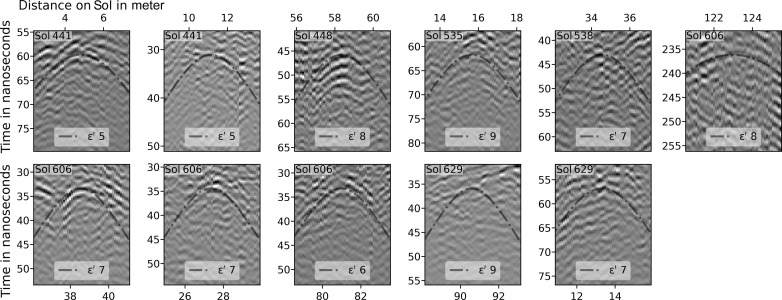
Diffraction Hyperbolas. Eleven RIMFAX radargram segments within delta sediments showing diffraction hyperbolas and best-fit hyperbolas and corresponding dielectric permittivities using a geometric ray tracing model (*15*). Sol locations are in the upper left corner.

Propagation velocities in the delta sediments were determined through the identification of 11 diffraction hyperbolas within the top 12 m of radargrams due to the presence of scatterers on Sols 441, 448, 535, and 538 in the Hawksbill Gap region, and Sols 606 and 629 in the Cape Nukshak region (Fig. 7). The diffraction hyperbolas were compared to the results of a geometric ray tracing model that accounts for the height of the RIMFAX antenna above a homogeneous subsurface (*15*) to determine the best-fit velocities. The results are summarized in Table 1, which shows mean subsurface dielectric permittivities, diffractor depths, velocities, and inferred densities for delta sediments from this study compared to the same quantities for the crater floor materials (*15*). The delta sediments appear to have lower densities and show fewer and less pronounced diffracting internal heterogeneities than the crater floor units below. We use our derived average delta sediment propagation velocity of 0.113 m/ns at all depths for the radargrams displayed in Figs. 2 to 5. The range of uncertainties in our derived propagation velocities has no appreciable effects on our geologic interpretations of the contact.

**Table 1.** Delta and crater floor subsurface dielectric properties.

Subsurface material	Number of hyperbolas	Mean dielectric permittivity ε′	Mean diffractor depth (m)	Mean velocity (m/ns)	Inferred mean density ρ (g cm^−3^) using ε′ = 2.0^ρ^
Lower delta sediments	11	7.18 ± 1.32	3.12	0.113 ± 0.013	2.84
Crater floor	150	8.9 ± 3.2	1.9	0.10 ± 0.02	3.1

### Airwave identification

We have identified airwave signatures in the RIMFAX radargrams close to the delta front that can be attributed to the presence of strongly reflecting near-vertical topographic surfaces visible to the rover. These airwaves are an expected consequence of the lack of shielding of the RIMFAX antenna (*10*). Airwaves can be differentiated from subsurface reflections in the radargrams by their high-frequency character, and the fact that they can be fit with hyperbolas with propagation velocities equal to the vacuum speed of light. The radargrams shown in Figs. 2 and 3 include airwave signatures that we have traced to cliff faces visible at the delta front and are not interpreted to represent subsurface geologic structures.
